# Emerging emitters in the spotlight

**DOI:** 10.1093/nsr/nwac245

**Published:** 2022-11-03

**Authors:** Shu Tao

**Affiliations:** College of Urban and Environmental Sciences, Peking University, China; College of Environmental Science and Technology, Southern University of Science and Technology, China

Anthropogenic greenhouse gas emissions conduce primarily to global warming. The Sixth Assessment Report of the Intergovernmental Panel on Climate Change has indicated that global warming is expected to hit 1.5°C in almost all emission scenarios in the early 2030s [1]. With risks ahead, global nations share both the mitigation responsibility and the adaptation challenge.

Global climate change mitigation has long focused on major emitters with either large economies or large populations. However, although historically their emissions have been great, most of the developed economies decoupled their carbon emissions and economic growth with the decarbonizing industry, and peaked their emissions by the first decade of this century [2]. Since more countries have proposed net-zero emission plans [3,4], the major emitters, including China and India, will reach peak emissions within the next decade. The countries from the ‘Rest of the World’ regions constitute the remaining uncertainty of global climate governance. In this issue, Cui *et al.* [5] investigated the energy-related CO_2_ emissions of ‘emerging emitters’, which refers to countries with faster-than-average emission growth excluding China and India, to determine their emission surge and explore their future possibilities.

Major emitting countries with large economies and populations have been the focus of mitigation research, literature and databases. Cui *et al.* [5] focus on emerging economies that have long been neglected in the field of carbon emissions and propose a scientific trend prediction, deepening the interpretation of global climate change emission reduction. On the other hand, as Cui *et al.* [5] propose, the emerging emitters vary in drivers of emissions, thus future work should focus on the heterogeneous and specific emission changes and mitigation pathways of the emerging emitters. This research contributes to the understanding, projections and data sets for previously ignored emerging economies in the field of climate change mitigation.

The trade-off between economic growth and emission reduction will remain a grand challenge for emerging emitters, while, as the authors estimate, the relatively high cost of mitigation technology hinders the emerging emitters’ emission abatement. Therefore, their work advocates international assistance for these emerging emitters and reveals the important role of historically major emitters in addressing global climate change, not only to provide technical and financial assistance to reduce emissions, but also to prioritize their own emission reduction agenda. More attention to emission reduction in emerging economies should be encouraged with this research.

When looking into the least-developed countries, the most basic scientific research, such as robust and verifiable energy and emission inventories, is what is most needed to contribute to global climate change mitigation. Although theoretically these countries emit much less CO_2_, they appear to be more sensitive and susceptible to climate change, and it is very difficult for academics to conduct thorough studies in order to understand their emission baselines and track their climate change mitigation efforts. Future work on fundamental data sets for emerging emitters will help to deepen the understanding of, and policy support for, the less-discussed regions of the world. New methods and techniques, combined with multisource data, including energy statistics, ground-based monitoring and satellite remote sensing, are required but need leadership and capacity-building from China and other major economies.

***Conflict of interest statement*.** None declared.
